# Inhibition of TLR4 Signaling Impedes Tumor Growth in Colitis-Associated Colon Cancer

**DOI:** 10.3389/fimmu.2021.669747

**Published:** 2021-05-07

**Authors:** Eva Pastille, Tabea Faßnacht, Alexandra Adamczyk, Nhi Ngo Thi Phuong, Jan Buer, Astrid M. Westendorf

**Affiliations:** Institute of Medical Microbiology, University Hospital Essen, University of Duisburg-Essen, Essen, Germany

**Keywords:** Toll-like receptor 4, colitis-associated colon cancer, inflammation, TAK-242, intestinal immune cells

## Abstract

Patients suffering from ulcerative colitis are at increased risk of developing colorectal cancer. Although the exact underlying mechanisms of inflammation-associated carcinogenesis remain unknown, the intestinal microbiota as well as pathogenic bacteria are discussed as contributors to inflammation and colitis-associated colon cancer (CAC). In the present study, we analyzed the impact of TLR4, the receptor for Gram-negative bacteria derived lipopolysaccharides, on intestinal inflammation and tumorigenesis in a murine model of CAC. During the inflammatory phases of CAC development, we observed a strong upregulation of *Tlr4* expression in colonic tissues. Blocking of TLR4 signaling by a small-molecule-specific inhibitor during the inflammatory phases of CAC strongly diminished the development and progression of colonic tumors, which was accompanied by decreased numbers of infiltrating macrophages and reduced colonic pro-inflammatory cytokine levels compared to CAC control mice. Interestingly, inhibiting bacterial signaling by antibiotic treatment during the inflammatory phases of CAC also protected mice from severe intestinal inflammation and almost completely prevented tumor growth. Nevertheless, application of antibiotics involved rapid and severe body weight loss and might have unwanted side effects. Our results indicate that bacterial activation of TLR4 on innate immune cells in the colon triggers inflammation and promotes tumor growth. Thus, the inhibition of the TLR4 signaling during intestinal inflammation might be a novel approach to impede CAC development.

## Introduction

The development of colorectal cancer is a major complication in patients suffering from ulcerative colitis (UC) (1). The duration and anatomic extent of disease, as well as the degree of inflammation are key factors that determine the risk of colorectal cancer in UC patients (2). Nevertheless, a decline in the incidence of cancer among UC patients has been observed in recent years (3), probably due to improved primary prevention and treatment, which underlines the importance of new therapeutic approaches. Currently, different medications are available that aim at controlling inflammation in UC. They are effectively used to attenuate intestinal inflammation and maintain remission in most UC patients, but their potential to reduce the patients´ subsequent risk for dysplasia and cancer remains unclear (2).

Chronic intestinal inflammation, as well as the development of colorectal cancer have been associated with alterations of the gut microbiome (4, 5). Toll-like receptors (TLRs) represent the link between the intestinal cell compartment and commensal bacteria, and are expressed either constitutively or inducible by immune cells, myofibroblasts, endothelial cells and epithelial cells of the gastrointestinal tract (6). As pattern-recognition receptors, TLRs detect conserved molecular products of microorganisms and their stimulation induces signaling pathways that result in initiation of immune responses (7). Thus, intestinal TLR signaling is pivotal for gut homeostasis (8, 9). In the gastrointestinal tract, characterization of TLR expression was mainly conducted in intestinal epithelial cells (IECs). In contrast to the small intestine, where IECs express relative low levels of various TLRs, IECs of the colon display high levels of TLR1, TLR2, TLR3, TLR4 and TLR5 expression at steady-state (10). Most of these TLR signaling pathways participate in the progression of UC and colorectal cancer, indicated by their altered expression during intestinal inflammation and tumorigenesis (11, 12). Furthermore, TLR4 signaling seems to be of central importance in the pathogenesis of colitis-associated colon cancer (CAC), as TLR4 was shown to be overexpressed in colonic IECs of UC patients but also in human and murine inflammation-associated colorectal neoplasia (13, 14). Moreover, experiments in different mouse models indicated that either TLR4 deletion protected *TLR4^-/-^* mice from tumor development or epithelial overexpression of TLR4 promoted inflammatory responses and tumorigenesis in CAC (15, 16). Thus, we aimed to explore the potential of TLR4 modulation by a specific TLR4 inhibitor during CAC in more detail and focused on its impact on lamina propia mononuclear cells, as preceding studies largely neglected TLR4 expression on immune cells.

In the current study, we show that acute DSS colitis affects TLR4 expression on innate immune cells as well as on epithelial cells in the colon. The inhibition of TLR4 signaling by TAK-242, a small-molecule-specific inhibitor of TLR4 signaling (17), during dextran sulfate sodium salt (DSS)-induced colitis attenuates intestinal inflammation without any overt side effects. Consequently, TAK-242 treatment impedes tumorigenesis of CAC, probably by preventing infiltration of macrophages. Therefore, the application of TAK-242 might be an approach to reduce the risk of dysplasia and cancer development in colitis patients.

## Materials and Methods

### Mice

Six to eight week old female BALB/c mice were purchased from ENVIGO (Horst, Netherlands) and maintained under specific pathogen-free conditions at the Animal Facility of the University Hospital Essen. All experiments were performed in accordance with the ethical principles and guidelines for scientific experiments and were approved by the local Landesamt für Natur-, Umwelt- und Verbraucherschutz (LANUV, North-Rhine-Westphalia, Germany).

### Induction of Acute DSS Colitis and Colitis-Associated Colon Cancer

BALB/c mice received 4% dextran sulfate sodium salt (DSS; MP Biomedicals, Heidelberg, Germany; MW, 36-50 kDa) in the drinking water for 7 days to induce acute colitis. Colitis-associated colon cancer was induced in BALB/c mice as described previously (18). In brief, mice were injected i.p. with 12.5 mg/kg body weight Azoxymethane (AOM, Sigma-Aldrich, Munich, Germany) followed by 2 to 3 cycles of 3% DSS supplemented drinking water.

### Disease Activity Index and Murine Colonoscopy

The magnitude of DSS-induced intestinal inflammation was assessed by a validated scoring system consisting of loss of body weight (score 1, 1–5%; score 2, 6–10%; score 3, 11–15%; score 4, 16–20%), rectal bleeding (score 0, no blood; score 2, blood visible; score 4, gross bleeding) and stool consistency (score 0, normal; score 2, loose stool; score 4, diarrhea). The obtained scores were summarized to a disease activity index (DAI) with a range of 0-12 (19). Numbers and sizes of tumors were determined in the distal part of the colon by murine colonoscopy. Therefore, mice were anesthetized by i.p. injection of ketamine and xylazine, and a rigid endoscope (Coloview miniendoscopic system, Karl Storz, Tuttlingen, Germany) was inserted up to the first flexure under visual control. Tumor sizes were graded on a scale of 1–5 according to the following sizes: size 1 (very small but detectable tumor), size 2 (tumor covering up to one eighth of the colonic circumference), size 3 (tumor covering up to a quarter of the colonic circumference), size 4 (tumor covering up to half of the colonic circumference), and size 5 (tumor covering more than half of the colonic circumference) (20). Tumor development was scored by the total number of tumors and the size of tumors.

### In Vivo TLR4 Inhibition and Antibiotic Treatment

TAK-242 (Cayman Chemical, *via* Biomol GmbH, Hamburg, Germany), a small-molecule TLR4 inhibitor that disrupts TLR4 interaction with adaptor molecules TIRAP and TRAM (17), was applied to BALB/c mice during DSS treatment. Mice were treated with 3 mg/kg TAK-242 i.p. every other day for 4 (acute DSS colitis) to 5 (DSS cycles during CAC induction) injections. To deplete the commensal microbiota, mice were orally treated with an antibiotic cocktail (Abx) including 1 mg vancomycin, 2 mg neomycin, 2 mg ampicillin (all Carl Roth GmbH, Karlsruhe, Germany), and 2 mg metronidazole (Sigma-Aldrich) per day and mouse. Efficient depletion was confirmed by quantification of microbial DNA in feces. In brief, stool samples were collected from mice, the weight was measured and then frozen at -80°C until further analysis. DNA from stool samples was extracted using the ZymoBIOMICS DNA Miniprep Kit (Zymo Research, Freiburg, Germany) according to the manufacturer´s recommendations.

### Colon Explant Culture and Cytokine Quantification

Colons were flushed with PBS and cut open longitudinally. A small explant (approximately 15-20 mg) from the distal part of the colon was cultured for 6 h in 300 µl of IMDM complete (IMDMc) culture medium supplemented with 10% heat-inactivated FCS, 25 mM β-Mercapthoethanol and antibiotics (100 U/ml penicillin, 0.1 mg/ml streptomycin). Cytokine levels in the supernatants were quantified by Luminex technology (R&D Systems, Abingdon, UK) on a Luminex 200 instrument. Cytokine concentrations were calculated using the Luminex IS software (Luminex Corporation, Austin, TX), and were normalized to the respective colon weight.

### Isolation of Intestinal Epithelial Cells and Lamina Propia Mononuclear Cells From the Colon

Intestinal epithelial cells (IELs) were isolated from the colon. Therefore, colons were incubated in 10 ml preheated PBS containing 1 mM Dithiothreitol (Carl Roth GmbH) under constant shaking for 10 min at 37°C. After washing the colons with PBS, they were transferred into 20 mL preheated Hanks’ Balanced Salt Solution (HBSS, Sigma-Aldrich) supplemented with 0,25 mM EDTA and incubated for 15 min under constant shaking at 37°C. Single IELs were gained by vigorous shaking and subsequent centrifugation. Afterwards, colonic lamina propia mononuclear cells (LPMCs) were isolated following a previously described protocol (18), without repeated EDTA incubation.

### Stimulation of Colonic Lamina Propia Mononuclear Cells With LPS and TAK-242 *In Vitro*

Lamina propia mononuclear cells (LPMCs) were isolated from the colon of naïve BALB/c mice and stained with antibodies against CD45 (clone 30-F11, BioLegend). CD45^+^ cell populations were sorted from the LPMCs using a FACSAria II cell sorter (BD Biosciences). CD45^+^ LPMCs were further seeded at 2x10^5^ cells in 200 µl of IMDMc medium in 96-well plates and stimulated with 1 µg/ml lipopolysaccharide (LPS, Sigma-Aldrich) in the presence or absence of 1 µM TAK-242. After 18 h, cell culture supernatants were collected to determine cytokine secretion *via* Luminex technology and resident cells were analyzed for TLR4 expression by flow cytometry.

### CT26 Culture and Cell Growth

The murine colon carcinoma cell line CT26 was obtained from the American Type Culture Collection (ATCC). CT26 cells were maintained in IMDMc culture medium in a humidified 5% CO_2_ atmosphere at 37°C. Before culture for growth analysis, cells were passaged twice. Afterwards, CT26 cells were analyzed for TLR4 expression by flow cytometry and then seeded at 1 x 10^4^ or 1 x 10^5^ cells in 1 ml of IMDMc medium in 6-well plates as triplicates. Cells were cultured in the presence or absence of 1 µM TAK-242 or an antibiotic cocktail (Abx, including 1 mg/ml vancomycin, neomycin, ampicillin and metronidazole). After 24 h, 48 h, and 72 h the amount of viable CT26 cells per well was determined.

### Quantification of Gene Expression by Real-Time PCR

Total RNA was extracted from colonic biopsies using the RNeasy Fibrous Tissue Mini Kit (Qiagen, Hilden, Germany). RNA was reversely transcribed into cDNA by using M-MLV reverse transcriptase (Promega, Walldorf, Germany) and a mixture of oligo(dT) and random hexamer primers (Invitrogen/ThermoFisher Scientific). Quantitative real-time PCR analysis were performed with the Fast SYBR Green Master Mix (ThermoFisher Scientific) and primers specific for *Tlr4* (Fwd: 5’-TGC TGG GGC TCA TTC ACT CAC-3’; Rev: 5’-ACA CTC AGA CTC GGC ACT TAG CAC-3’). Quantitative real-time PCR reactions were run on an ABI PRISM Sequence Detection System (Applied Biosystems/ThermoFisher Scientific). Relative gene expression levels were calculated with included standard curves for each gene and further normalization to the housekeeping gene *Rps9* (Fwd: 5’-CTG GAC GAG GGC AAG ATG AAG C-3’; Rev: 5’-TGA CGT TGG CGG ATG AGC ACA-3’).

### Antibodies and Flow Cytometry

Cells isolated from the colon were incubated with fluorochrome-labeled antibodies against CD4 (RM4-4), Ly6G (1A8), Siglec-F (E50-2440) (all BD Biosciences, Heidelberg, Germany), CD8 (53-6.7), CD19 (1D3), CD45 (30-F11), CD49b (DX5), TLR4 (SA15-21), CD11c (HL3) (all BioLegend, San Diego, CA), CD11b (M1/70), F4/80 (BM8), EpCAM (G8.8) and Foxp3 (FJK-16s) (all eBioscience/ThermoFisher Scientific, Langenselbold, Germany). Dead cells were stained with the fixable viability dye eFluor 780 (ThermoFisher Scientific). Intracellular staining for Foxp3 was performed with the eBioscience™ Foxp3/Transcription Factor Staining Buffer Set (ThermoFisher Scientific) according to the manufacturer’s protocol. Stained cells were analyzed using a FACS LSRII and DIVA Software (BD Biosciences).

### Statistical Analysis

All statistical analyses were calculated using GraphPad Prism 7.03 software (GraphPad, La Jolla, CA). To test for normal distribution, D’Agostino and Pearson omnibus normality test was used. Single comparison of normally distributed data was performed by paired or unpaired Student’s *t*-test. Differences between means of more than two normally distributed groups were assessed by either one-way or two-way ANOVA followed by Tukey´s, Sidak´s or Dunnett´s post-test. When means of more than two not normally distributed groups were compared, the Kruskal-Wallis test followed by Dunn´s post-test was used. Correlation was calculated using Spearman correlation analysis. Statistical significance was set at the levels of *, p≤ 0.05; **, p≤ 0.01 and ***, p≤ 0.001.

## Results

### *Tlr4* Expression Is Upregulated in the Colon During the Course of Colitis-Associated Colon Cancer

**Figure 1:**
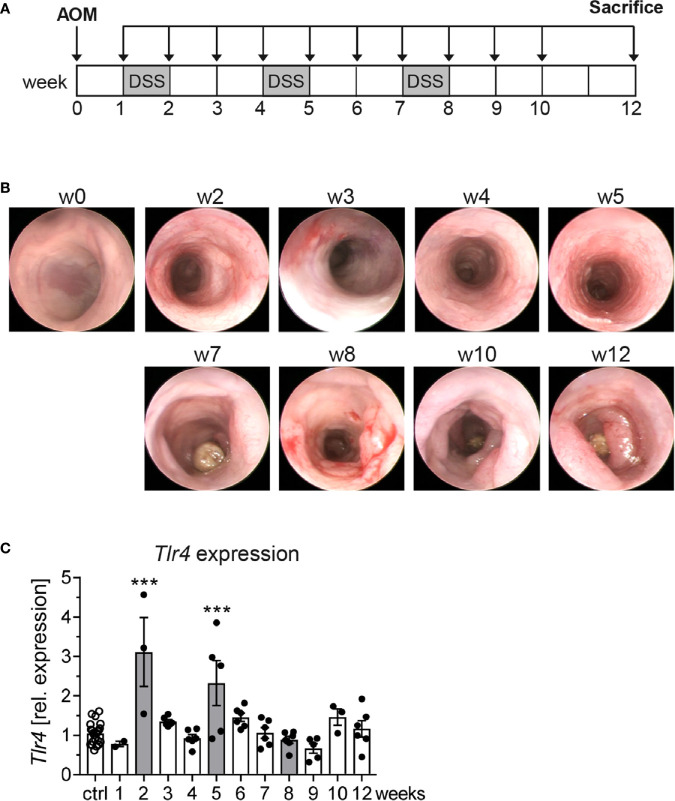
*Tlr4* expression in colonic biopsies during the course of colitis-associated colon cancer. **(A)** Schematic treatment protocol of colitis-associated colon cancer (CAC) induction in BALB/c mice. After an intraperitoneal injection of the procarcinogen azoxymethane (AOM), three seven-day cycles of dextran sulfate sodium (DSS) were given *via* the drinking water. Mice that received normal drinking water served as control group (ctrl). **(B)** At indicated time points, the tumor development in AOM/DSS treated mice was analyzed by murine colonoscopy and **(C)** *Tlr4* expression in colonic biopsies was determined *via* qRT-PCR. Bars represent the mean ± SEM of data from two independent experiments with n=22 ctrl and n=3-6 CAC mice per time point. Differences of *Tlr4* expression between AOM/DSS treated mice and the ctrl group were calculated using one-way ANOVA followed by Dunnett´s post-test (***p ≤ 0.001).

Strong expression of TLR4 in tumors of colorectal cancer patients has been associated with poor prognosis (21). Given that TLR4 signaling is also involved in intestinal homeostasis and its activation may cause chronic inflammation (22), we aimed to investigate the role of TLR4 in a murine model of colitis-associated colon cancer (CAC). We used the AOM/DSS model, in which repeated inflammatory insults initiated by DSS promote tumor development (**Figure 1A**). Using murine colonoscopy, clear signs of inflammation, e.g. bleeding mucosa, altered vascular pattern, or abundant fibrin were evident at week 2, 3, 5 and 8 after DSS treatment. First dysplastic changes could be detected at week 4, while tumor development significantly increased from week 7 to week 12 (**Figure 1B**). When *Tlr4* expression was analyzed in colonic biopsies, we observed a significant colonic upregulation of *Tlr4* at week 2 and 5 in AOM/DSS-treated mice compared to control mice, concurrently with the first two inflammatory phases. Interestingly, *Tlr4* expression in the colon did not increase at later time points, neither after the last DSS cycle nor during tumor progression (**Figure 1C**).

### Colonic CD45^+^ Leukocytes Display Enhanced TLR4 Expression During DSS-Induced Inflammation

**Figure 2:**
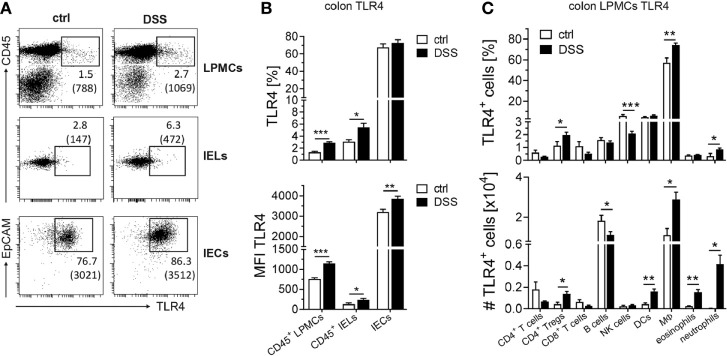
Expression of TLR4 in the colonic epithelium and the lamina propia during acute colitis. BALB/c mice were treated with DSS in the drinking water to induce an acute colitis. Control mice (ctrl) received normal drinking water. At day seven, mice were sacrificed and cells from the lamina propia or intestinal epithelial cells were isolated from the colon. **(A, B)** CD45^+^ lamina propia mononuclear cells (LPMCs), CD45^+^ intestinal epithelial lymphocytes (IELs), and CD45^-^ EpCAM^+^ intestinal epithelial cells (IECs) were analyzed for TLR4 expression *via* flow cytometry. **(A)** Representative dot plot of TLR4 expression among the indicated cell types in the colon of ctrl and DSS mice. Numbers indicate percentages and the MFIs (in brackets) of TLR4 expression. **(B)** Summarized data of the percentages (upper panel) and the MFI (lower panel) of TLR4 expression from two independent experiments with n=7 ctrl and n=10 DSS mice is shown as mean ± SEM. **(C)** CD45^+^ LPMCs were further subdivided into Foxp3^-^ CD4^+^ T cells, CD8^+^ T cells, Foxp3^+^ CD4^+^ Tregs, CD19^+^ B cells, DX5^+^ NK cells, CD11c^+^ F4/80^-^ dendritic cells (DCs), CD11c^+^ F4/80^+^ macrophages (MΦs), CD11b^+^ Siglec-F^+^ eosinophils and CD11b^+^ Ly6G^+^ neutrophils, and TLR4 expression was analyzed. Absolute numbers of TLR4^+^ cells were calculated. Data from two independent experiment with n=5 ctrl mice and n=11 DSS mice are shown as mean ± SEM. Statistical analyses were performed by unpaired Student´s t-test (*p ≤ 0.05; **p ≤ 0.01; ***p ≤ 0.001).

In recent studies, considerable TLR4 expression in the colon was reported for intestinal epithelial cells (10), likewise in patients suffering from acute colitis, in which epithelial TLR4 expression is further upregulated during inflammation (13). In accordance, colonic *Tlr4* expression in AOM/DSS-treated mice significantly increased during the first two DSS cycles. To confirm that colonic epithelial cells are the cellular source of *Tlr4* expression during DSS-induced inflammation, we isolated intestinal epithelial cells (IECs) and intestinal epithelial lymphocytes (IELs), as well as lamina propria mononuclear cells (LPMCs) from the colon of mice suffering from acute DSS colitis and analyzed TLR4 expression by flow cytometry. As expected, in the colon of healthy control mice the majority of IECs expressed TLR4, but no increase in the frequency of TLR4^+^ IECs could be observed after DSS treatment. Nevertheless, we detected higher MFIs of TLR4 on IECs from DSS mice indicating further increase of TLR4 expression on IECs during inflammation. Surprisingly, also CD45^+^ leukocytes, isolated from the intestinal epithelium and the lamina propia of mice suffering from DSS-induced colitis, displayed enhanced TLR4 expression (**Figures 2A, B**). Among the CD45^+^ LPMC population, notable TLR4 expression was found on macrophages, and to a lesser extent on DCs and NK cells. All other analyzed immune cells showed rather low levels of TLR4 expression (**Figure 2C**, upper panel). DSS-induced inflammation further augmented TLR4 expression on macrophages, but also frequencies of TLR4^+^ Ly6G^+^ neutrophils and TLR4^+^ Foxp3^+^ CD4^+^ Tregs were significantly elevated. During DSS-induced inflammation, neutrophils, eosinophils and Tregs infiltrate the colon, while the frequency of B cells is reduced. In terms of absolute numbers, TLR4^+^ macrophages, DCs, eosinophils, neutrophils and Tregs were significantly increased, while DSS treatment lowered the numbers of TLR4^+^ B cells in the colon (**Figure 2C**, lower panel).

### Inhibition of Microbial Sensing by TAK-242 or Antibiotic Treatment Impedes Tumor Development in Colitis-Associated Colon Cancer

**Figure 3:**
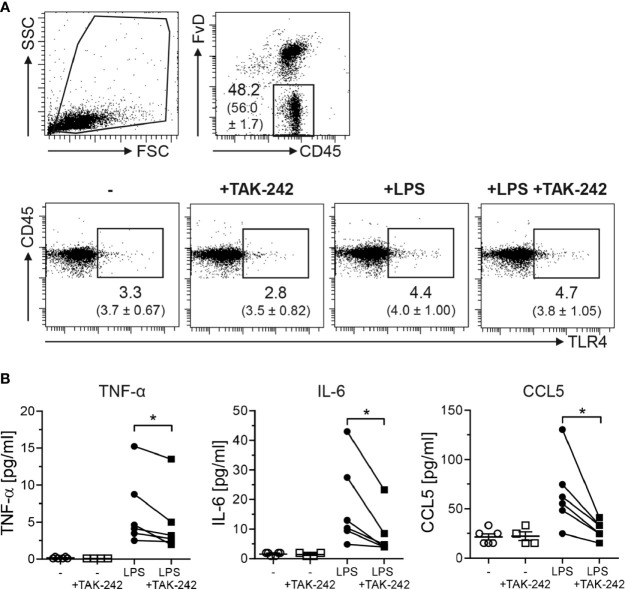
TAK-242 blocks LPS-induced production of pro-inflammatory cytokines. CD45^+^ LPMCs were isolated from the colon of BALB/c mice and stimulated for 18 h with LPS in the presence or absence of TAK-242. **(A)** After stimulation, CD45^+^ LPMCs were analyzed for TLR4 expression by flow cytometry. Representative dot plots show the percentage (mean ± SEM in brackets) of TLR4 of gated viable CD45^+^ LPMCs. **(B)** Concentrations of TNF-α, IL-6 and CCL5 were measured in the cell culture supernatants by Luminex technology. Graphs show the results as mean ± SEM from two independent experiments and differences between groups were calculated by paired Student’s *t*-test (*p≤ 0.05).

To elucidate the impact of TLR4 expression in the colon during the course of CAC, we made use of TAK-242 (resatorvid). First, we tested the efficiency of TLR4 inhibition by TAK-242 in isolated LPMCs. We stimulated sorted CD45^+^ cells from the colon of BALB/c mice *in vitro* with LPS in the presence or absence of TAK-242. After *in vitro* culture, around 50 - 60% of all cultured cells were still viable (**Figure 3A**, upper panel). Unstimulated CD45^+^ LPMCs expressed low levels of TLR4 on their surface, comparable to those TLR4 levels that were measured in CD45^+^ LPMCs which were directly isolated from the colon untreated BALB/c mice. TAK-242 treatment did not alter TLR4 expression in cultured CD45^+^ LPMCs. When CD45^+^ LPMCs were stimulated with LPS, irrespective if TAK-242 was present, CD45^+^ LPMCs slightly, but not significantly, upregulated TLR4 expression on the surface (**Figure 3A**, lower panel). Stimulation of CD45^+^ LPMCs with LPS strongly induced the secretion of pro-inflammatory cytokines, like TNF-α and IL-6, as well as the secretion of the chemokine CCL5. As expected, the incubation of CD45^+^ LPMCs with TAK-242 significantly blocked the LPS-induced production of these cytokines and chemokines (**Figure 3B**).

**Figure 4:**
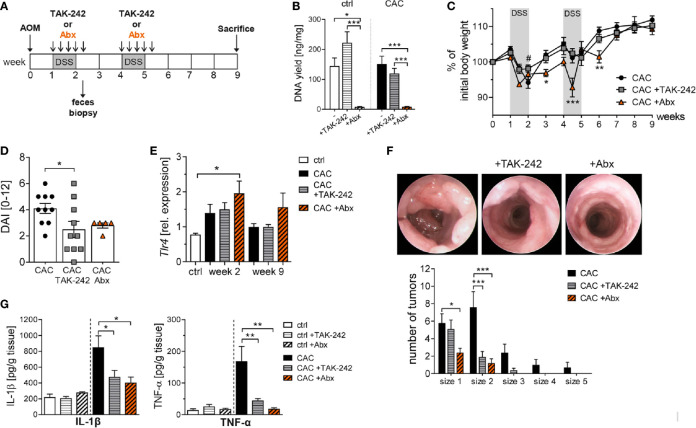
TAK-242 or antibiotic treatment alters tumor development in colitis-associated colon cancer. **(A)** Schematic treatment protocol of colitis-associated colon cancer (CAC) induction and TAK-242 or antibiotic (Abx) treatment in BALB/c mice. During DSS treatment mice received either i.p. injections of TAK-242 every second day (five injections in total) or orally received an antibiotic cocktail. Mice that received normal drinking water served as control group (ctrl). **(B)** At week 2, after the first DSS cycle and Abx treatment, feces were collected and the DNA yield was determined. The graph shows the results as mean ± SEM from two experiments (n=4 ctrl mice and n=10 CAC mice per group), and differences between groups were calculated using two-way ANOVA followed by Tukey´s post-test (*p≤ 0.05; ***p≤ 0.001). **(C)** Body weight of AOM/DSS-treated mice relative to initial body weight during the course of the experiment. The results from two experiments (n=2-7 ctrl mice and n=5-10 CAC mice per group) are shown as mean ± SEM and differences between groups were calculated using two-way ANOVA followed by Dunnett’s post-test (*p≤ 0.05; **p≤ 0.01; ***p≤ 0.001 CAC+Abx vs. CAC; ^#^p≤ 0.05 CAC+TAK-242 vs. CAC). **(D)** At week 2, the disease activity index (DAI) was assessed. **(E)** *Tlr4* expression in colonic biopsies was determined by RT-PCR at week 2 and at week 9. **(F)** At week 9, tumor development was analyzed by murine endoscopy and tumor numbers and sizes for each mouse were calculated. Representative endoscopic pictures from the colon of untreated, TAK-242-treated or Abx-treated CAC mice are shown. **(G)** Concentration of TNF-α and IL-1β were measured in the supernatants of colonic biopsies from CAC and ctrl mice by Luminex. **(D–G)** The graphs show data as mean ± SEM from two experiments (n=2-7 ctrl mice and n=5-10 CAC mice per group) and differences between groups were calculated using one-way ANOVA followed by Dunnett´s post-test (*p ≤ 0.05; **p ≤ 0.01; ***p ≤ 0.001).

Next, we applied TAK-242 *in vivo* in the mouse model of CAC and compared its effect with an antibiotic treatment which was already demonstrated to efficiently diminish inflammation-induced tumor development in the colon (23). Mice were treated repeatedly with 3mg/kg bodyweight TAK-242 i.p., or with an antibiotic cocktail (Abx) of metronidazole, vancomycin, ampicillin and neomycin during the cycles of DSS treatment (**Figure 4A**). The antimicrobial efficacy of Abx application was confirmed by a nearly complete absence of bacterial DNA in the feces of Abx treated mice (**Figure 4B**). Of note, Abx treatment had a substantial impact on the mice overall health status during both cycles of DSS administration, as indicated by a rapid loss of body weight, (**Figure 4C**). In contrast, CAC mice that received TAK-242 during DSS treatment lost less body weight compared to untreated CAC mice at week 2 (**Figure 4C**). Determining the magnitude of intestinal inflammation by scoring rectal bleeding, stool consistency and body weight, we observed a substantial decrease in the disease activity index (DAI) scores in mice treated with TAK-242 or Abx (**Figure 4D**). When we analyzed *Tlr4* expression after the first DSS cycle at week 2, we found increased levels of *Tlr4* in the colon of all CAC groups. Neither TAK-242 treatment, nor Abx treatment in CAC mice reduced colonic *Tlr4* expression. During tumor progression, no alteration in the expression of *Tlr4* was observed in CAC and TAK-242 treated CAC mice compared to healthy control mice, while Abx treated CAC mice displayed a slight but not significant upregulation of colonic *Tlr4* expression (**Figure 4E**). Most important, TAK-242 or Abx treatment significantly reduced the number and the growth of tumors as detected by endoscopic analysis at week 9 (**Figure 4F**). Appropriately, colon biopsies from TAK-242 and Abx treated mice secreted considerably lower amounts of tumor-promoting cytokines, such as IL-1β and TNF-α (**Figure 4G**).

### TAK-242 Treatment Has No Direct Impact on Tumor Cell Growth

**Figure 5:**
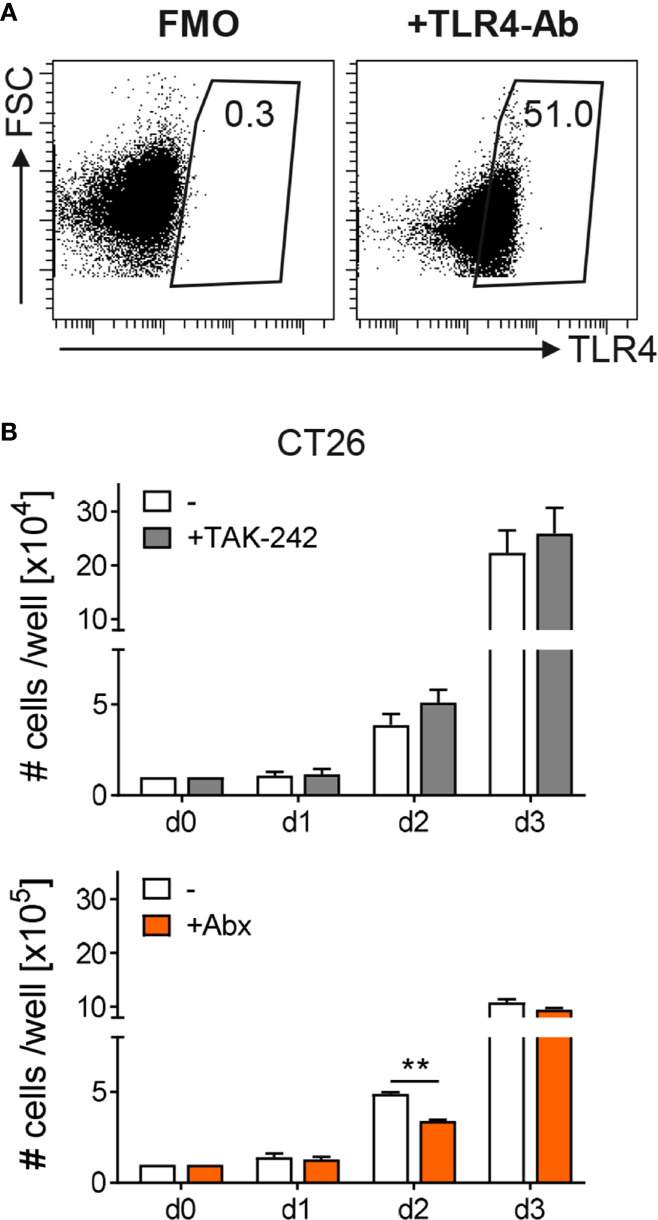
Influence of TAK-242 or antibiotic treatment on tumor cell growth. The murine colon cancer cell line CT26 was stained with or without (FMO) antibodies against TLR4 and TLR4 expression was evaluated by flow cytometry. Representative dot plots show the percentage of TLR4 on gated viable CT26 cells. Numbers indicate percentages of TLR4 expression. CT26 cells were further cultured in the absence or presence of TAK-242 (as 1 x 10^4^ cells per well at day 0) or Abx (as 1 x 10^5^ cells per well at day 0). After indicated time points CT26 cells were harvested and counted. Graphs show the results as mean ± SEM from two independent experiments and differences between groups were calculated using two-way ANOVA followed by Sidak´s post-test (**p ≤ 0.01).

To further determine how TAK-242 or Abx modulate tumor development, we first tested their influence on tumor cell growth. Therefore, the murine colon cancer cell line CT26 was cultured *in vitro* in the absence or presence of TAK-242 or the antibiotic cocktail of metronidazole, vancomycin, ampicillin and neomycin and growth development was monitored for 3 days. Flow cytometry analysis revealed that CT26 cells express TLR4 on their surface (**Figure 5A**). Nevertheless, culturing CT26 cells with TAK-242 had no significant impact on their growth *in vitro.* In contrast, the presence of Abx slightly reduced the growth of CT26 cells, at least at day 2 of cell culture (**Figure 5B**).

### Reduced Tumor Growth After TAK-242 or Antibiotic Treatment Is Associated With Low Frequencies of Tumor-Infiltrating Macrophages

**Figure 6:**
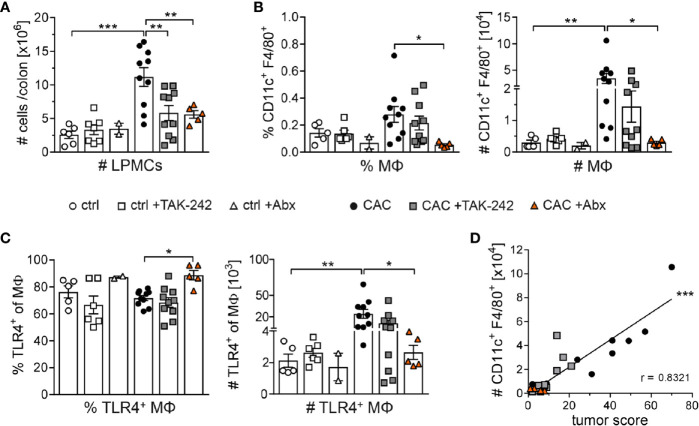
Distribution of macrophages in the colon after TAK-242 or antibiotic treatment. Colitis-associated colon cancer was induced in BALB/c mice. Mice either received i.p. injections of TAK-242 or received an Abx cocktail of vancomycin, neomycin, ampicillin and metronidazole. Mice that received normal drinking water served as control group (ctrl). **(A)** At week 9, lamina propia mononuclear cells (LPMCs) were isolated from the colon and counted to determine absolute cell numbers. **(B)** Frequencies and absolute numbers of CD11c^+^F4/80^+^ macrophages (MΦ) and **(C)** TLR4^+^ MΦ among isolated LPMCs were assessed by flow cytometry. The graphs show data as mean ± SEM from two experiments (n=2-7 ctrl mice and n=5-10 CAC mice per group) and differences between groups were calculated using one-way ANOVA or Kruskal-Wallis test followed by Dunnett´s or Dunn`s post-test (*p≤ 0.05; **p≤ 0.01; ***p≤ 0.001). **(D)** Correlation between the absolute numbers of colonic CD11c^+^F4/80^+^ MΦ and the tumor score was calculated using Spearman correlation analysis. Lines represent linear regression of correlation with Spearman r coefficient (***p≤ 0.001).

As macrophages are the immune cells in the lamina propia with the strongest TLR4 expression, we further investigated the impact of TAK-242 and Abx treatment on macrophages. Of note, CAC is associated with a strong infiltration of myeloid cells into the lamina propia (24). Mice suffering from AOM/DSS induced CAC exhibited higher LPMCs counts than ctrl mice. Remarkably, both TAK-242 and Abx application were linked with lower absolute numbers of LPMCs in the colon of CAC mice (**Figure 6A**). Macrophages, gated as CD11c^+^ F4/80^+^ cells, accumulated in the colon due to CAC induction. Whereas the inhibition of TLR4 by TAK-242 in CAC mice reduced percentages and absolute numbers of colonic macrophages only in tendency, Abx treatment completely abrogated accumulation of macrophages in the colon after CAC induction (**Figure 6B**). Interestingly, while the percentage of TLR4^+^ macrophages in the colon of CAC mice was slightly enhanced after Abx treatment, the absolute numbers of TLR4^+^ macrophages in TAK-242 and Abx treated CAC mice were reduced (**Figure 6C**). Finally, the absolute number of CD11c^+^ F4/80^+^ macrophages in the colon of all CAC groups positively correlated with the tumor score (**Figure 6D**), pointing towards a connection between infiltrating macrophages and tumor development in CAC.

### TAK-242 and Antibiotic Treatment Alleviate Intestinal Inflammation in DSS-Induced Colitis

**Figure 7:**
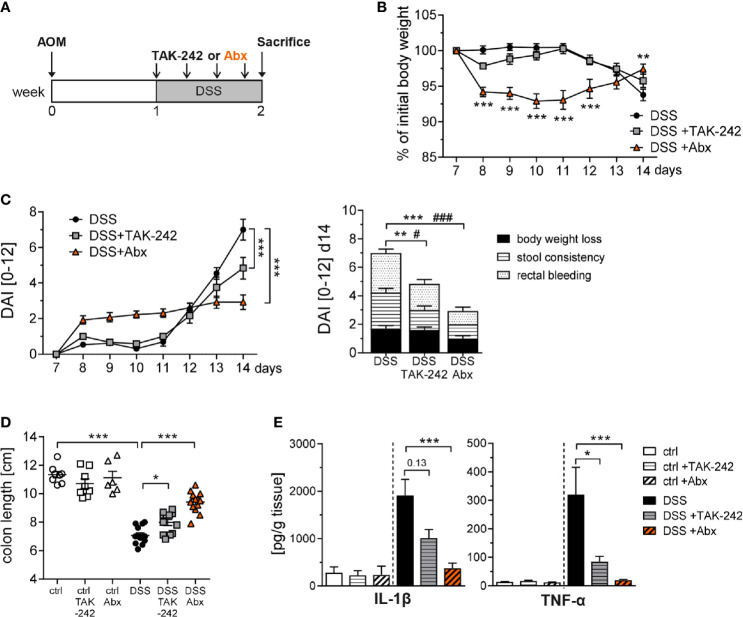
TAK-242 or antibiotic treatment alleviates intestinal inflammation in DSS-induced colitis. **(A)** Schematic treatment protocol of DSS colitis induction and TAK-242 or Abx treatment in BALB/c mice. During DSS treatment mice received either i.p. injections of TAK-242 every second day (four injections in total) or were treated with an Abx cocktail of vancomycin, neomycin, ampicillin and metronidazole (six days in total). Mice that received normal drinking water served as control group (ctrl). **(B)** Body weight and **(C)** disease activity index (DAI) was measured during DSS treatment. Results are expressed as body weight or DAI over the course of the experiment and the DAI was subdivided into clinical parameters at week 2. The results from three experiments (n=9-13 DSS mice per group) are shown as mean ± SEM and differences between groups were calculated using two-way ANOVA followed by Dunnett´s post-test (***p≤ 0.001) or by two-way ANOVA followed by Tukey´s post-test (DAI at d14, **p≤ 0.01; ***p≤ 0.001 for differences between stool consistency; ^#^p≤ 0.05; ^###^p≤ 0.001 for differences between rectal bleeding). **(D)** At week 2, colons were prepared and the length was measured. **(E)** Biopsies from colon samples were cultured *in vitro* and secretion of IL-1β and TNF-α in the supernatants were determined by Luminex. Bars show the mean ± SEM of data from three experiments (n=6-8 ctrl mice and n=12-13 DSS mice per group). Statistical significance was calculated using Kruskal-Wallis test followed by Dunn´s post-test (*p≤ 0.05; **p≤ 0.01; ***p≤ 0.001).

TLR4 inhibition and Abx treatment during the inflammatory phases of CAC potently decreased tumor growth, probably through the reduction of chronic inflammation. The diminished DAI after DSS administration gave a first hint in this direction. To pursue this assumption, we subjected BALB/c mice to the AOM/DSS regimen, applied TAK-242 or Abx, and subjected the mice to extensive analysis in week 2 (**Figure 7A**). Mice that received Abx rapidly lost body weight but were able to fully recover within 7 days of DSS treatment and even displayed an improved body weight compared to untreated DSS mice at day 14. In contrast, mice injected with TAK-242 started to loose body weight rather late but were also protected from serious body weight loss (**Figure 7B**). When we analyzed the DAI over time, Abx-treated DSS mice exhibited an early increase in DAI, which could be completely attributed to body weight loss within the first 5 days of DSS treatment. After 7 days of DSS treatment, mice injected with TAK-242, as well as Abx treated mice showed significantly alleviated signs of intestinal inflammation, as indicated by a reduction of rectal bleeding and diarrhea, and an overall decreased DAI (**Figure 7C**). Consequently, colon shortening was less pronounced after TLR4 inhibition and significantly improved after Abx application (**Figure 7D**). Secretion of the pro-inflammatory cytokines IL-1β and TNF-α was significantly reduced in the colon of TAK-242 or Abx treated DSS mice, further demonstrating attenuated intestinal inflammation (**Figure 7E**).

Taken together, our data reveal that reducing TLR signaling is an effective approach to alleviate DSS-induced intestinal inflammation with less side effects and thereby protecting mice from CAC development.

## Discussion

Typically, inflammation is a prerequisite to clear infections and heal wounds. According to Hanahan and Weinberg, unresolved or chronic inflammation enables the acquisition of cancer hallmark functions (25). In inflammatory bowel disease, such as UC, inflammation appears to result from aberrant host immune responses to the intestinal microbiota driven by both, genetic predisposition and environmental factors (26).

In this study, we identified TLR4 activation as a mechanism that mediates intestinal inflammation and thereby promotes tumor development. First, we observed a strong increase in *Tlr4* expression in the colon of mice during the inflammatory phases of AOM/DSS treatment, which points towards an involvement of TLR4 in colitis induction. Well in line with our data, TLR4 expression was shown to be significantly increased in intestinal epithelial cells (IECs) and in the lamina propia of the inflamed mucosa of UC patients (13, 27). Of note, we did not find a boost of intestinal *Tlr4* expression when tumor development set in. This is in contrast to studies of UC patients´ mucosa samples and murine colon samples, in which epithelial TLR4 expression was upregulated in dysplastic lesions and colitis-associated tumors (14, 15). A possible reason for this discrepancy might be the difference in TLR4 measurement. While Fukata et al. determined TLR4 expression on protein level by immunofluorescence and western blot and divided the colon tissue of CAC mice in non-dysplastic and tumor lesions, we analyzed *Tlr4* expression on mRNA level in whole colon biopsies of CAC mice. Thereby we eventually did not detect tumor cell-specific upregulation of TLR4 protein expression. Nevertheless, when we analyzed the cellular distribution of TLR4 in the colon of mice after the first DSS administration by flow cytometry in more detail, we found that a high percentage of IECs in healthy control and DSS mice express TLR4. As IECs and also CD45^+^ LPMCs and IELs, further increased TLR4 expression on their surface in response to inflammation, we conclude that, at least at week 2, upregulation of *Tlr4* expression in colonic biopsies reflects increased cellular TLR4 protein expression. This finding of high TLR4 expression on IECs coincides with recent data from Price et al., who investigated intestinal TLR patterns in reporter mice under steady state, and found TLR4 expression on both the apical and basolateral surfaces of IECs in the proximal colon, as well as intracellular TLR4 in colonic IECs. They concluded from this, that IECs could respond to TLR4 ligands that have translocated across the epithelium (10), an aspect that could be important when the epithelium displays barrier defects during inflammation. In contrary to Price et al., we could demonstrate an increase of epithelial TLR4 expression after the induction of DSS colitis. This may also underline the influence of different TLR4 measurement techniques or relies on the differences in the application of DSS to induce colitis.

Notably, our experiments revealed that macrophages in the colon displayed very strong TLR4 expression with a significant increase after DSS treatment. Likewise, inflammation-dependent induction of TLR4 expression was found in intestinal macrophages of UC patients (27). In contrast to colonic CD45^+^ LPMCs and especially macrophages, which upregulated TLR4 expression during DSS induced inflammation, *in vitro* stimulation of CD45^+^ LPMCs with LPS did not induce upregulation of cell-surface TLR4. Remarkably, neither TAK-242 treatment *in vitro* altered TLR4 expression on CD45^+^ LPMCs, nor TAK-242 treatment *in vivo* modulated *Tlr4* expression in colonic biopsies. Still, TAK-242 treatment *in vitro* efficiently inhibited LPS induced production of pro-inflammatory cytokines and chemokines in CD45^+^ LPMCs and *in vivo* TAK-242 reduced the release of TNF-α and IL-1β from colonic biopsies. We conclude from these results that the effectiveness of TAK-242 treatment is not associated with reduced TLR4 expression. Moreover, we speculate that the increase of TLR4 expression on LPMCs *in vivo* is not solely dependent on LPS, but must be induced by different signals. Indeed, TLR4 signaling can be regulated at multiple levels by negative regulators, such as Guanine nucleotide-binding protein α subunit-interacting vesicle-associated protein (GIV) (28, 29) or positive regulators. Cytokines, such as GM-CSF and IFN-γ (30–32), or stimulation of the EGFR receptor (33) were shown to provide positive signals that increase TLR4 expression on monocytes, macrophages or microglia cells. Interestingly, TLR4 signaling in IECs induces the expression and release of amphiregulin, which binds to the EGFR receptor in an autocrine and paracrine manner, subsequently stimulating proliferation of IECs (34). Thereby, IECs might not only stimulate their own TLR4 expression but ongoing proliferation could also promote tumor formation. However, whether other TLR4 expressing immune cells than IECs or macrophages are involved in inflammatory processes in the colon needs further investigation. Moreover, whether an increase of TLR4 expression on colonic IECs and CD45^+^ immune cells has any functional consequences, has yet to be clarified.

The overall importance of TLR4 signaling on acute colitis and CAC development has been intensely studied in murine models using TLR4^-/-^ mice and TLR4 inhibitors, such as TAK-242 or antagonist TLR4 antibodies (15, 24, 35). Although complete deletion of TLR4 in TLR4^-/-^ mice did not protect from severe DSS-induced inflammation (36), the specific lack of TLR4 signaling on colonic epithelial cells inhibited CAC development, as well as recruitment and activation of Cyclooxygenase (Cox)-2 expressing macrophages in the AOM/DSS model (14, 16). In agreement with these findings, we observed that TLR4 inhibition by TAK-242 during the onset of CAC was also potent to reduce macrophage infiltration and impede tumor growth. Contrary to the results from TLR4^-/-^ mice, others and we found that the inhibition of TLR4 signaling by TAK-242 during DSS treatment alleviated intestinal pathology (35).

Wang et al. reported that TAK-242 application in DSS colitis interfered with the JAK2/STAT3 pathway and significantly altered the intestinal microbiota (35). Regarding microbial composition, they observed a markedly decreased abundance of *Proteobacteria*, *Cyanobacteria* and *Epsilonbacteraeota*, while *Akkermansia* were strongly expanded in TAK-242 treated DSS mice compared to DSS mice. Notably, *Akkermansia (A.) muciniphila* was recently shown to blunt tumorigenesis in the AOM/DSS colon cancer model by expanding CTLs in the colon (37), and, as a mucin-degrading bacterium, it releases metabolites that provide energy for butyrate-producing intestinal bacteria (38). Butyrate has many beneficial roles, e.g. anti-inflammatory capacities, improving the gut barrier function, and prevention of colon cancer (39). Moreover, butyrate was also shown to induce TLR4 expression on colon cancer cells *in vitro* (40). Therefore, TAK-242 mediated alterations of the microbial composition and increased butyrate levels might modulate *Tlr4* expression and tumor development. Despite the fact that tumorigenesis in CAC is obviously connected to dysbiosis of the microbiota (23, 41), we did not observe changes in *Tlr4* expression at the late stage of CAC development. This might be dependent on the transition of IECs to tumor cells that display altered responses to microbial stimulation (42). Well in line with this, when we treated mice in an advanced tumor state of CAC (from week 6 to week 9) with TAK-242, we could not detect any differences in the overall number or size of colonic tumors compared to non-treated animals.

TAK-242 treatment during DSS colitis significantly reduced levels of pro-inflammatory and pro-tumorigenic TNF-α and IL-1β in the colon. These reductions in colonic cytokine secretion were still present in the colon of TAK-242 treated tumor-bearing mice. Given that CAC development is highly dependent on the extent of inflammation and inflammatory cytokines (43), we propose that TAK-242 affects tumor formation mainly *via* the inhibition of the inflammatory environment. Of note, cytokines, such as IL-6, or IL-1β, not only induce pathogen clearance, but promote the proliferation and survival of intestinal epithelial cells, as well as differentiation of pro-tumorigenic Th17 cells (44–46), thereby supporting tumorigenesis. Nevertheless, it remains to be specified which cells were the source of theses cytokines in our experiments. Analysis on the cellular composition in the colon of CAC mice revealed reduced frequencies of macrophages after TAK-242 treatment, indicating that macrophages might be the central target of TLR4 signaling. Classically activated macrophages produce high levels of pro-inflammatory cytokines, such as TNF-α, IL-1β, IL-6, IL-12, or IL-23, in response to TLR stimulation (47). In the course of intestinal inflammation, Ly6C^hi^ monocytes are recruited to the gut mucosa, but their differentiation into mature macrophages that maintain immune homeostasis is disrupted (48). Immature monocyte-like Ly6C^int^ macrophages that retain their pro-inflammatory capacity through the secretion of inflammatory cytokines, including IL-1β, accumulate and aggravate tissue damage (49). During tumorigenesis, accumulation of tumor-associated macrophages (TAMs) is widely observed (50). Generally, TAMs are associated with poor prognosis in solid tumors as they acquire an immunosuppressive, pro-angiogenic, and pro-metastatic phenotype (50). In the murine AOM/DSS model of CAC, the contribution of macrophages to tumorigenesis was corroborated by specifically blocking the CCR2-dependent recruitment of macrophages, which was sufficient to prevent tumor development (24, 51). In addition to the general impact of CCR2 blockade, Yang et al. further found that targeting the sensing of microbiota by the oral administration of Abx or the TLR4 inhibitor TAK-242 was potent in reducing the recruitment and activation of macrophages during CAC. Simultaneously, Abx or TAK-242 treatment of CAC mice potently reduced tumor numbers (24). These findings are well in line with our results, as we also observed significantly diminished tumor development after TAK-242 or Abx treatment, accompanied with lower frequencies of colonic macrophages. While Abx application seemed to be superior in restraining CAC, the use of Abx should be well considered due to unwanted side effects. Besides a rapid and strong loss of body weight, a decline in certain metabolites of the intestinal microbiota could be a consequence of prolonged Abx exposure. Depending on their function, these microbial metabolites, either are involved in the suppression of inflammation and cancer (e.g. short-chain fatty acids), or promote carcinogenesis (e.g. secondary bile acids) (52). Although our experiments created similar results in terms of tumor development, Yang et al. used a different regimen of Abx or TAK-242 application. Antibiotics were administered in the drinking water before the onset of CAC and TAK-242 was given together with LPS. Furthermore, TLR4 expression was only measured on colonic epithelial cells. Therefore, Yang et al. propose that the microbiota-induced activation of TLR4 on IECs mediated CCL2 production and thereby facilitated the recruitment of macrophages with a monocyte-like inflammatory phenotype (24). We, on the contrary, speculate that Abx or TAK-242 might directly act on TLR4-expressing intestinal macrophages to block their cytokine production and thus, limit inflammation-induced carcinogenesis. Moreover, we propose an advantage in applying TAK-242 or Abx during DSS administration as this would mimic a therapeutic approach in UC patients. Still, additional research would be reasonable to uncover the direct effect of TAK-242 on intestinal macrophages and the inhibition of tumor growth.

Overall, our results strengthen the importance of TLR4 activation during intestinal inflammation for the development of colitis-associated colon cancer. Targeting TLR4 signaling by TAK-242 has the potential to reduce innate immune cell infiltration and pro-inflammatory mediators in the colon with a long-lasting impact on tumor growth which could be a benefit for the treatment of UC patients.

## Data Availability Statement

The original contributions presented in the study are included in the article/supplementary material. Further inquiries can be directed to the corresponding author.

## Ethics Statement

The animal study was reviewed and approved by Landesamt für Natur-, Umwelt- und Verbraucherschutz North-Rhine-Westphalia, Germany.

## Author Contributions

TF, AA, and NN performed the experiments and analyzed the data. EP designed and conducted the experiments, analyzed the data and wrote the manuscript. JB was involved in data discussion and in drafting the manuscript. AW designed and supervised the study and wrote the manuscript. All authors contributed to the article and approved the submitted version.

## Funding

This work was supported by the German Research Foundation (DFG) research grant GRK1949 to AW and JB and PA 2792/2-1 to EP. We acknowledge support by the Open Access Publication Fund of the University of Duisburg-Essen.

## Conflict of Interest

The authors declare that the research was conducted in the absence of any commercial or financial relationships that could be construed as a potential conflict of interest.
